# Pediatric Dosing of Intravenous Sotalol Based on Body Surface Area in Patients with Arrhythmia

**DOI:** 10.1007/s00246-017-1683-9

**Published:** 2017-07-28

**Authors:** Xiaomei Li, Yan Zhang, Haiju Liu, He Jiang, Haiyan Ge, Yi Zhang

**Affiliations:** Department of Pediatric Cardiology, Heart Center, The First Hospital of Tsinghua University, School of Clinical Medicine, Tsinghua University, Beijing, 100016 People’s Republic of China

**Keywords:** Intravenous sotalol, Tachyarrhythmias, Intravenous dosing, Pediatric dosing

## Abstract

In a recently published study, we evaluated the efficacy and safety of intravenous sotalol in pediatric patients with incessant tachyarrhythmias and we have found that intravenous sotalol is effective and safe. Our dosing regimen was based on the body weight of the patients. In the US, the recommendation for intravenous sotalol dosing in pediatric patients is based on body surface area (BSA) while taking into consideration the patients’ age. The purpose of this paper is to show the correspondence of a body weight-based dosing regimen when expressed for BSA as mg/m^2^. We evaluated the similarity of a body weight-based dose to that calculated based on BSA using the US labeling recommendations. Of the 83 patients, 5 were newborns (age: 0–30 days), 39 infants and toddlers (age: 1–24 month), 26 young children (age: >2–6 years), 11 older children (age: 6–12 years), and 2 adolescents (age: 14 years). Each received a loading dose of 1 mg/kg intravenous sotalol administered over 10 min followed by a maintenance dose of 4.5 mg/kg/day. There was a close correlation between the sotalol loading doses calculated based on body weight and BSA across the entire age range (*r* = 0.977, *p* < 0.001). In most of the age groups, the body weight-based loading doses were lower or equal to the BSA-based doses. Only in the adolescents were the body weight-based doses higher. The maintenance doses given in our study were significantly higher than the BSA-based dose in newborns: 75 ± 6 versus 53 ± 8 mg/m^2^, *p* < 0.05; infants/toddlers: 88 ± 14 versus 77 ± 7 mg/m^2^, *p* < 0.001; younger children: 113 ± 12 versus 85 mg/m^2^, *p* < 0.001; older children: 123 ± 16 versus 85 mg/m^2^, *p* < 0.01; and adolescents 157 ± 30 versus 85.5 mg/m^2^. Despite the rapid administration of the loading dose and the increased maintenance doses, our body weight-based dosing regimen was safe. Only one newborn had significant adverse event (AV block) that resolved spontaneously after discontinuation of the infusion.

## Introduction

Sotalol is a potent antiarrhythmic agent with class III antiarrhythmic and beta-blocking properties. Sotalol is often used in the treatment of pediatric arrhythmias with good results [1–6]. Until recently, the English literature has described the efficacy predominantly of the oral sotalol formulation in pediatric patients. However, oral sotalol has a slow onset and it is not practical to use in urgent or emergency situations for the termination of hemodynamically compromising or life-threatening tachyarrhythmias. Intravenous sotalol is available in the United States. Using intravenous sotalol instead of oral administration is advisable in emergency situation, because therapeutic blood level can be achieved faster, as well as the intravenous administration overcomes the uncertainty of variable oral absorption of the drug from the gastrointestinal track. In a recently published study, we evaluated the efficacy and safety of intravenous sotalol in pediatric patients with incessant tachyarrhythmias and we have found that intravenous sotalol is effective and safe [7]. To our best knowledge, this is the first and only publication regarding intravenous use of sotalol in a pediatric population in the English literature. We employed a dosing regimen, which was based on the body weight of the patients. In the United States, the recommendation for intravenous sotalol dosing in pediatric patients is based on body surface area (BSA) [8]. The purpose of this paper is to compare body weight-based dosing regimen to sotalol dosing expressed in terms of BSA as mg/m^2^. This may aid physicians who use BSA-based dosing to evaluate the results of our clinical study and to plan for the use of intravenous sotalol in their patients. We provide a comparison between our dosing regimen and the dosing regimen recommended by the FDA in the approved labeling for pediatric patients in the United States.

## Materials and Methods

The intravenous sotalol dosing regimen, patients’ characteristics, and arrhythmia type, as well as efficacy and safety of intravenous sotalol, were described [7]. Briefly, a total of 83 pediatric patients with persistent tachycardias were given intravenous sotalol. These patients did not have structural heart disease and all had normal cardiac function (ejection fraction ≥50%). There were five newborn babies with ages between 0 and 30 days (mean age: 24 days, range 10–30 days), there were 39 infants and toddlers with ages between 1 month and 2 years (mean age: 8.5 months, range 1.2–24 months), and there were 37 children with ages between 2 and 12 years. Of these children, there were 26 younger children with ages between 2 and 6 years (mean age: 3.9 years, range 28 months to 6 years) and 11 older children with ages between 6 and 12 years (mean age: 9 years, range 6.4–12 years), and there were 2 adolescents, both 14 years old. Thirty patients had atrioventricular reentrant tachycardia, 36 had atrial tachycardia, 9 had atrial flutter, 3 had atrial fibrillation, and 5 had ventricular tachycardia. The intravenous dosing regimen consisted of a loading dose of 1 mg/kg body weight sotalol administered over 10 min followed by a maintenance dose of 4.5 mg/kg/day.

For the purpose of this paper, body surface area was calculated for each child using the Mosteller formula: BSA (m^2^) = SQRT ([Height (cm) × Weight (kg)]/3600). Then the administered loading dose and maintenance dose were divided by the BSA to calculate the equivalent dose in mg/m^2^ BSA for each child.

The recommended starting dose of oral sotalol is based on body surface area in the US and calculated as 30 mg/m^2^ × BSA × Age factor. The age factor applies to children up to 2 years of age and is provided in a nomogram in the product label [8]. The bioavailability of oral sotalol is approximately 95%. To count for the less than 100% bioavailability, the intravenous dose is 95% of the oral dose to deliver the same amount with intravenous administration as with the oral dose. For the purpose of comparison of our body weight-based doses to the recommended BSA-based dosing, we calculated the BSA-based intravenous sotalol dose as 30 mg/m^2^ × BSA × Age factor × 0.95 [8].

Statistical analysis was performed using IBM SPSS version 22 software package (IBM Corporation, Armonk, NY, USA). Data are presented as mean ± standard deviation along with the range. Each continuous variable was tested for normal distribution using the Kolmogorov–Smirnov and Shapiro–Wilk tests of normality. The body weight- and BSA-based doses did not show normal distribution. Therefore, a nonparametric test was performed (Wilcoxon signed-rank test) for comparison. The correlation between the two dosing methods was evaluated by linear regression analysis using a Pearson correlation coefficient. A *p* value of <0.05 was considered statistically significant.

## Results

The loading doses of intravenous sotalol for each of the five age groups are shown in Table 1. These are the doses that we employed in our previously published study. The loading doses were calculated as 1 mg/kg body weight “rounded up” to the nearest 0.5 mg. The equivalent doses expressed as mg/m^2^ BSA are also shown in the table under the column heading of “Loading Dose/BSA.” The recommended starting dose in the US is 30 mg/m^2^ BSA above 2 years of age. As shown in Table 1, the body weight-based doses approximate the BSA doses in children and adolescents. In newborn babies and infants, the doses were less than 30 mg/m^2^ since an age factor is used in the calculation of the BSA-based dose, and the result is a lower dose than 30 mg/m^2^.

**Table 1:** Loading dose of intravenous sotalol in each age group calculated as 1 mg/kg body weight (mg) and the equivalent dose expressed in terms of body surface area (mg/m^2^)

Age groups	Age	Loading dose (mg)	Loading dose/BSA (mg/m^2^)
Newborns (*n* = 5) (0–30 days)	24 ± 7.2 days (10–30 days)	3.9 ± 0.7 (3–5)	16.3 ± 1.4 (14.5–18.8)
Infants/toddlers (*n* = 39) (1–24 months)	8.5 ± 7.1 months (1.2–24 months)	7.6 ± 3 (3–13)	19.5 ± 3 (12–24.4)
Younger children (*n* = 26) (2–6 years)	3.9 ± 1.2 years (2.3–6 years)	17.3 ± 5.4 (11–34)	25 ± 2.7 (20.5–33.1)
Older children (*n* = 11) (6–12 years)	9.0 ± 1.9 years (6.4–12 years)	28 ± 7.6 (19–40)	27.3 ± 3.6 (21.4–33)
Adolescents (*n* = 2) (12–18 years)	14 ± 0 years (14–14 years)	55.5 ± 11.2 (48–63)	34.8 ± 6.3 (32.5–39)

Data are given as mean ± standard deviation (range). Sotalol doses were calculated as 1 mg/kg body weight (dose in mg) and the equivalent doses were expressed for body surface area (BSA) by dividing each dose in each patient by the BSA (mg/m^2^)

**Fig. 1:**
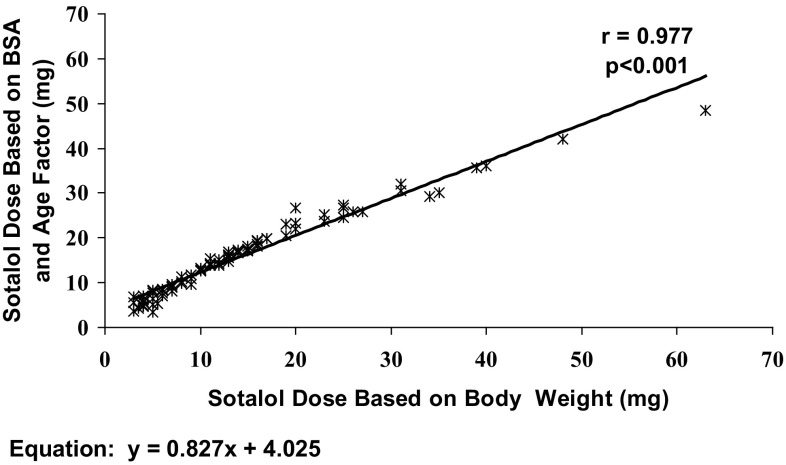
Correlation between sotalol loading doses calculated based on body weight and body surface area employing an age factor

We evaluated the agreement between the doses that were given in our study based on 1 mg/kg body weight and the doses that patients would have been given if the doses were calculated based on BSA using the US labeling recommendation for starting dose. The results are shown in Fig. 1 and Table 2. As shown in Fig. 1, there is a close correlation between the sotalol doses calculated by the two methods (*r* = 0.977, *p* < 0.001) across the entire age range. The corresponding doses of those calculated based on body weight and BSA are also shown for each age group in Table 2. The body weight-based sotalol doses were lower than the BSA-based doses in newborn babies, infants, and toddlers, as well as in young children. In older children, the loading doses were practically equal by both methods, while in adolescents the body weight-based doses were higher than the BSA-based doses. Thus, in most age groups, body weight-based doses were less than or equal to the BSA-based doses. We administered the loading dose over 10 min, while the recommendation in the US is to administer the dose over 5 h.

**Table 2:** Loading dose of intravenous sotalol in each age group calculated as 1 mg/kg body weight (mg) and expressed for body surface area (BSA) versus sotalol doses calculated as 30 mg/m^2^ BSA employing an age factor

Age groups	Age	Loading dose (mg/m^2^ BSA)	
		Calculated based on body weight	Calculated based on BSA (label recommended)
Newborns (*n* = 5) (0–30 days)	24 ± 7.2 days (10–30 days)	16.3 ± 1.4 (14.5–18.8)	17.6 ± 2.5 (12.7–19.2)
Infants/toddlers (*n* = 39) (1–24 months)	8.5 ± 7.1 months (1.2–24 months)	19.5 ± 3 (12–24.4)	25.5 ± 2.5** (19.2–28.5)
Younger children (*n* = 26) (2–6 years)	3.9 ± 1.2 years (2.3–6 years)	25 ± 2.7 (20.5–33.1)	28.5 ± 0** (28.5–28.5)
Older children (*n* = 11) (6–12 years)	9.0 ± 1.9 years (6.4–12 years)	27.3 ± 3.6 (21.4–33)	28.5 ± 0 (28.5–28.5)
Adolescents (*n* = 2) (12–18 years)	14 ± 0 years (14–14 years)	34.8 ± 6.3 (32.5–39)	28.5 ± 0 (28.5–28.5)

Data are given as mean ± standard deviation (range). Sotalol doses were calculated based on body weight (1 mg/kg) and expressed for body surface (BSA) by dividing the dose in mg by BSA m^2^. For comparison, doses calculated based on body surface area (BSA) following the recommendation in the product’s US label

**p < 0.001

The maintenance dose of intravenous sotalol in each of the five age groups is shown in Table 3. The intravenous sotalol dose was calculated as 4.5 mg/kg/day. The doses were “rounded up” to the nearest 0.5 mg and administered as a continuous infusion. The equivalent doses have been expressed for BSA and are shown under the column heading of “Maintenance Dose/BSA” in mg/m^2^. The US recommendation for initial maintenance dose is to administer the starting dose three times a day. Thus, in patients above 2 years of age the recommended daily oral dose is 90 mg/m^2^. Body weight-based dosing exceeds the BSA-based dosing in certain age groups (younger children, older children, and adolescents).

**Table 3:** Maintenance dose of intravenous sotalol in each age group calculated as 4.5 mg/kg body weight (mg) and the equivalent dose expressed for body surface area (mg/m^2^)

Age groups	Age	Maintenance dose (mg)	Maintenance dose/BSA (mg/m^2^)
Newborns (*n* = 5) (0–30 days)	24 ± 7.2 days (10–30 days)	18.0 ± 3 (14–23)	75.1 ± 6.4 (67.5–86.4)
Infants/toddlers (*n* = 39) (1–24 months)	8.5 ± 7.1 months (1.2–24 months)	34.1 ± 13.4 (14–60)	87.9 ± 13.5 (56–112.5)
Younger children (*n* = 26) (2–6 years)	3.9 ± 1.2 years (2.3–6 years)	78.3 ± 2 4.3 (50 to 153)	112.9 ± 12 (93 to 148.8)
Older children (*n* = 11) (6–12 years)	9.0 ± 1.9 years (6.4–12 years)	126.2 ± 33.6 (86–176)	123 ± 15.6 (98.5–149.4)
Adolescents (*n* = 2) (12–18 years)	14 ± 0 years (14–14 years)	250 ± 49.5 (216–284)	156.6 ± 30.2 (146.1–167.1)

Data are given as mean ± standard deviation (range). Sotalol doses were calculated as 4.5 mg/kg body weight (dose in mg) per day and the equivalent doses expressed for body surface area (BSA) by dividing each dose in each patient by BSA (mg/m^2^)

**Fig. 2:**
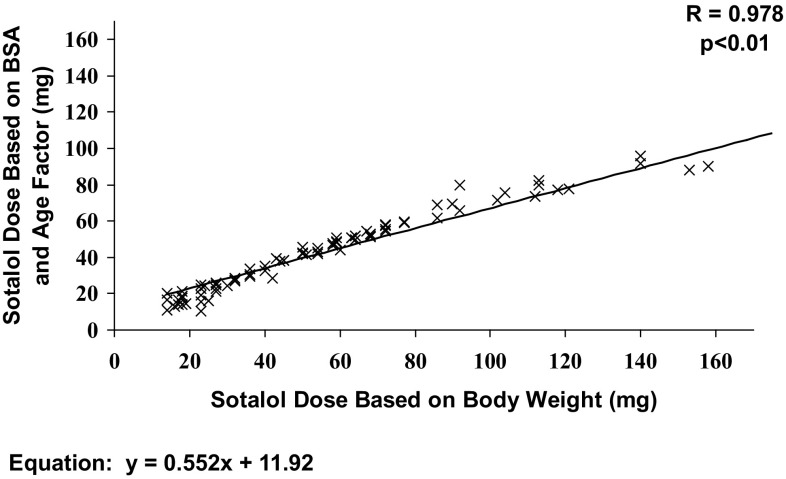
Correlation between sotalol maintenance doses calculated based on body weight and body surface area employing an age factor

We have evaluated the corresponding doses between the maintenance doses that were given in our study and the doses that patients would have been given if the dosing was calculated using BSA as recommended in the US label. There is a close correlation of the sotalol doses calculated by the two methods (*r* = 0.978, *p* < 0.001) (Fig. 2). However, as seen from the slope of the trend line, the body weight-based doses are higher. The doses as would be calculated by the two computational methods are shown for each age group in Table 4. The body weight-based maintenance doses are higher than the BSA-based doses for each age group.

**Table 4:** Maintenance dose of intravenous sotalol in each age group calculated as 4.5 mg/kg body weight (mg) per day and expressed for body surface area (BSA) versus sotalol doses calculated as daily 90 mg/m^2^ BSA employing an age factor

Age groups	Age	Maintenance/daily dose (mg/m^2^ BSA)	
		Calculated based on body weight	Calculated based on BSA (label recommended)
Newborns (*n* = 5) (0–30 days)	24 ± 7.2 days (10–30 days)	75.1 ± 6.4 (67.5–86.4)	52.7 ± 7.6* (38–57.7)
Infants/toddlers (*n* = 39) (1–24 months)	8.5 ± 7.1 months (1.2–24 months)	87.9 ± 13.5 (56–112.5)	76.6 ± 7.4*** (57.7–85.5)
Younger children (*n* = 26) (2–6 years)	3.9 ± 1.2 years (2.3–6 years)	112.9 ± 12 (93 ± 148.8)	85.5 ± 0.0*** (85.5–85.5)
Older children (*n* = 11) (6–12 years)	9.0 ± 1.9 years (6.4 to 12 years)	123 ± 15.6 (98.5–149.4)	85.5 ± 0.0** (85.5–85.5)
Adolescents (*n* = 2) (12–18 years)	14 ± 0 years (14–14 years)	156.6 ± 30.2 146.1–167.1)	85.5 ± 0.0 (85.5–85.5)

Data are given as mean ± standard deviation (range). Maintenance dose calculated based on body weight: Sotalol doses were calculated based on body weight (4.5 mg/kg/day) and expressed for body surface area (BSA) by dividing the dose in mg by BSA m^2^. For comparison, doses calculated based on body surface area (BSA) following the recommendation in the product’s US label

*p < 0.05, **p < 0.01, ***p < 0.001

## Discussion

There are limited data about the efficacy and safety of sotalol dosing regimens in pediatric patients. We have proposed an intravenous dosing regimen based on body weight and published the efficacy and safety of this dosing regimen [7]. Since in the US pediatric dosing of sotalol is based on BSA [8], we compared body weight-based dosing to BSA-based dosing. The loading doses of intravenous sotalol calculated based on body weight are less than or equal to the loading doses calculated based on BSA as recommended in the US label. Only in adolescents are the body weight-based loading doses higher. The maintenance doses employed in our study were significantly higher than those calculated from the US label. It is not uncommon that a loading dose of intravenous sotalol is calculated based on body weight and administered over a short period of time in adult patients [9–14]. Examples are 1 mg/kg administered over 10 min [9, 10], 1.5 mg/kg administered over 30 min [11, 12], and 1.5 mg/kg administered over 10 min [13, 14]. Doses of 0.5 mg/kg to 1.5 mg/kg can be safely administered over a 10-min period for the management of acute arrhythmias and can be repeated at 6-h intervals if necessary according to regulatory authority labeling outside the US [15]. This dosing recommendation applies to adults with no recommendation for pediatric patients. Knudson and coworkers employed BSA-based dosing in newborn babies and infants (age under 24 months) without employing an age factor [3]. They administered a median dose of 152 mg/m^2^/day sotalol (range 65–244) to 78 patients with supraventricular tachycardia (62% were neonates, 46% had congenital heart disease) after the patients failed at least one other antiarrhythmic agent. If they would have followed the dosing recommendation in the US label, the median dose of sotalol would have been 57 mg/m^2^/day (range 27–88 mg/m^2^/day) which is roughly one-third of the median dose that they administered. In 90% of the patients, the supraventricular tachycardias were controlled without adverse events. Läer and coworkers set out to develop a safe and effective pediatric oral sotalol dosing regimen [4]. The authors chose a body weight-based dose calculation. They recommended 2 mg/kg/day starting dose and 4 mg/kg/day target dose for neonates, 3 mg/kg/day starting dose and 6 mg/kg/day target dose for infants and children <6 years, and 2 mg/kg/day starting dose and 4 mg/kg/day target dose for children >6 years. They noted an increased QT prolongation in neonates.

We have found the intravenous dosing regimen we utilized to be effective in a variety of supraventricular and ventricular arrhythmias. Sixty percent of patients were converted to sinus rhythm with intravenous sotalol alone and an additional 15% were converted when sotalol was combined with intravenous propafenone, resulting in a 75% conversion rate [7]. Administering a loading dose over 10 min has the advantage of achieving therapeutic sotalol blood levels faster. Sotalol can prolong the rate-corrected QT interval (QTc) and the QTc prolongation is dose proportional and correlates well with sotalol blood concentration [16–18]. Thus, rapid loading of sotalol results in higher blood concentration than a slow infusion and may result in significant QTc prolongation and proarrhythmia. QTc prolongation above 500 ms is a marker for the risk of developing torsade de pointes ventricular tachycardia, especially when the left ventricular function is decreased [8]. In our study, the average QTc interval increased by 34 ms, but only two patients developed clinically significant QTc prolongation (480 and 500 ms, respectively), which resolved after discontinuing the intravenous sotalol and continuing the therapy with oral sotalol. Of the 83 cases, we observed only 1 significant adverse event (AV block). We believe that selecting patients with normal left ventricular function (EF >50%) and normal QTc before drug administration was the reason for the excellent safety profile. We also recommend frequent measurement of QT interval, heart rate, and blood pressure while administering intravenous sotalol to ensure safety.

These findings support the use of intravenous sotalol administered on a mg/kg basis, as well as the use of their corresponding doses of intravenous sotalol based on BSA. Future research on the use of intravenous sotalol to control arrhythmias in children, as well as the use of intravenous sotalol to prevent JET and other arrhythmias following congenital heart surgery, is suggested.
